# Effects of *Syzygium aromaticum*-Derived Triterpenes on Postprandial Blood Glucose in Streptozotocin-Induced Diabetic Rats Following Carbohydrate Challenge

**DOI:** 10.1371/journal.pone.0081632

**Published:** 2013-11-22

**Authors:** Andile Khathi, Metse R. Serumula, Rene B. Myburg, Fanie R. Van Heerden, Cephas T. Musabayane

**Affiliations:** 1 School of Laboratory Medicine and Medical Sciences, University of KwaZulu-Natal, Durban, South Africa; 2 School of Chemistry and Physics, University of KwaZulu-Natal, Durban, South Africa; Broad Institute of Harvard and MIT, United States of America

## Abstract

**Purpose:**

Recent reports suggest that the hypoglycaemic effects of the triterpenes involve inhibition of glucose transport in the small intestine. Therefore, the effects of *Syzygium* *spp*-derived triterpenes oleanolic acid (OA) and maslinic acid (MA) were evaluated on carbohydrate hydrolyzing enzymes in STZ-induced diabetic rats and consequences on postprandial hyperglycaemia after carbohydrate loading.

**Methods:**

We determined using Western blot analysis the expressions of α-amylase and α-glucosidase and glucose transporters SGLT1 and GLUT2 in the small intestine intestines isolated from diabetic rats treated with OA/MA for 5 weeks. *In* *vitro* assays were used to assess the inhibitory activities of OA and MA against α-amylase, α-glucosidase and sucrase.

**Results:**

OA and MA ameliorated postprandial hyperglycemia in carbohydrate loaded diabetic rats as indicated by the significantly small glucose area under the curve (AUC) in treated diabetic animals compared with that in untreated diabetic rats. Western blotting showed that OA and MA treatment not only down-regulated the increase of SGLT1 and GLUT2 expressions in the small intestine of STZ-induced diabetic rats, but also inhibited small intestine α-amylase, sucrase and α-glucosidase activity. IC_50_ values of OA against α-amylase (3.60 ± 0.18 mmol/L), α-glucosidase (12.40 ± 0.11 mmol/L) and sucrase (11.50 ± 0.13 mmol/L) did not significantly differ from those of OA and acarbose.

**Conclusions:**

The results of suggest that OA and MA may be used as potential supplements for treating postprandial hyperglycemia.

**Novelty of the Work:**

The present observations indicate that besides improving glucose homeostasis in diabetes, OA and MA suppress postprandial hyperglycaemia mediated in part via inhibition of carbohydrate hydrolysis and reduction of glucose transporters in the gastrointestinal tract. Inhibition of *α*-glucosidase and *α*-amylase can significantly decrease the postprandial hyperglycaemia after a mixed carbohydrate diet and therefore can be an important strategy in the management of postprandial blood glucose levels in NIDDM patients.

## Introduction

Postprandial hyperglycemia ascribed to hydrolysis of carbohydrates by digestive enzymes in the small intestine is a major risk factor for complications in diabetic patients [1-3]. Small intestine α-glucosidase and pancreatic α-amylase are the key enzymes of dietary carbohydrate digestion in humans. Glucose released upon carbohydrate digestion is absorbed from the intestinal lumen into the blood mainly via sodium-dependent glucose co-transporter (SGLT1), localized to the enterocyte apical or brush-border membrane (BBM), and the basolateral facilitative glucose transporter 2 (GLUT2) [4]. Evidence obtained from experimental diabetes indicates that the capacity of the small intestine to absorb glucose in diabetes increases mainly due to enhanced activity and abundance of SGLT1 and GLUT2 [5]. Thus, controlling postprandial glucose level is critical during early treatment of diabetes mellitus to avert postprandial glucose excursions thereby reducing chronic vascular complications [6]. The duration of postprandial hyperglycaemia and magnitude of glucose concentration trigger oxidative stress-linked diabetic complications [7]. 

Commercially available synthetic inhibitors of carbohydrate hydrolyzing enzymes are effective in retarding carbohydrate hydrolysis and glucose absorption to suppress postprandial hyperglycaemia [8]. Quercetin, a flavonoid antioxidant, isolated from medicinal plants blunts postprandial hyperglycaemic spike via inhibition of carbohydrate hydrolysis [9,10]. Furthermore, *α*-glucosidase inhibitors with increased potency and lesser adverse effects than the existing drugs have also been isolated from medicinal plants [11]. We have reported that the hypoglycaemic properties of plant-derived oleanolic acid (OA) are in part mediated via inhibition of glucose transport in the small intestine [12], but effects on key carbohydrate hydrolyzing enzymes remain unanswered. Therefore, the present project was designed to evaluate the effects of *Syzygium* *spp*-derived triterpenes oleanolic acid (OA) and maslinic acid (MA) on carbohydrate hydrolyzing enzymes in normal and diabetic rats and consequences on postprandial hyperglycaemia associated with polysaccharide, disaccharide and monosaccharide challenge. We also evaluated the effects of OA and MA on the expression of α-amylase and α-glucosidase as well as on glucose transporters SGLT1 and GLUT2 in the small intestine to establish whether these triterpenes had direct effects on carbohydrate digestion and glucose transport in the small intestine. 

## Materials and Methods

### Drugs and chemicals

Drugs were sourced from standard pharmaceutical suppliers. All other chemicals which were of analytical grade quality were purchased from standard commercial suppliers. 

### Extraction methods

OA and MA were isolated from *Syzygium aromaticum* [(Linnaeus) Merrill & Perry] [Myrtaceae] (cloves) using a standard protocol that has been validated in our laboratory [13,14]. Air-dried *S. aromaticum* flower buds (500 g) were milled and sequentially extracted twice at 24 h intervals at room temperature using 1 L dichloromethane (DCM), and ethyl acetate (720 ml) on each occasion. Subsequently, the extract was concentrated under reduced pressure at 55 ± 1 °C using a rotary evaporator to yield dichloromethane solubles (DCMS) and ethyl acetate solubles (EAS). The EAS containing mixtures of oleanolic/ursolic acid and methyl maslinate/methyl corosolate were purified by silica gel 60 column chromatography with hexane: ethyl acetate solvent systems, 7:3 for OA and 6:4 for MA. This yielded OA and MA, respectively which were further purified by recrystallization from chloroform-methanol (1:1, v/v). The structures of OA and MA were confirmed by spectroscopic analysis using 1D and 2D, ^1^H and ^13^C nuclear magnetic resonance (NMR) spectroscopic experiments.

### Animals

Male Sprague-Dawley rats (250-300 g) maintained on free access to standard rat chow (Meadows, Pietermaritzburg, South Africa) and water *ad libitum* were used throughout the study. They were maintained in standard environmental conditions with 12h light/12h dark cycle. All animal protocols were reviewed and approved by the University of KwaZulu-Natal animal ethics committee.

### Induction of diabetes mellitus

Experimental type 1 diabetes mellitus was induced in male Sprague-Dawley rats using a previously described protocol [15]. Briefly, the animals were administered a single intraperitoneal injection of 60 mg/ kg STZ in freshly prepared 0.1 M citrate buffer (pH 6.3). Control group received the vehicle, citrate buffer through the same route. Animals that exhibited glucosuria after 24 h, tested by urine strips (Rapidmed Diagnostics, Sandton, South Africa) were considered diabetic. Seven days later, the blood glucose concentration of STZ-induced diabetic rats greater than 20 mmol L^-1^ was considered as stable diabetes. 

### Experimental design

The effects of OA/MA on postprandial blood glucose concentration and intestinal carbohydrate-hydrolyzing enzymes and glucose transporters were examined in non-diabetic and STZ-induced diabetic male Sprague-Dawley rats. The inhibitory activities of OA and MA against carbohydrate hydrolyzing enzymes were studied *in vitro*. 

### Oral glucose tolerance (OGT) responses

The rats were divided into the following groups: control and treated non-diabetic rats and control and treated STZ-induced diabetic rats (n = 6 in each group). After an 18 h fasting period, glucose was measured (time 0) followed by loading with monosaccharide (glucose; 0.86 g/kg, p.o), disaccharide (sucrose; 1.72 g/kg, p.o.) or polysaccharide (starch; 0.086 g/kg, p.o) delivered into the stomach by a gavage needle (18-gauge, 38 mm long curved, with a 21/4 mm ball end). To determine the effects of triterpenes on postprandial glucose, separate groups of animals were administered OA and MA (80 mg/kg) dissolved in dimethyl sulfoxide and deionized water. The selection of these doses was based on the posology from previous studies in our standard laboratory [16]. Rats treated with DMSO/water (3 ml/kg, p.o.) and standard drugs acarbose (100 mg/kg, p.o.) and phlorizin (100 mg/kg, p.o.) served as untreated and positive controls, respectively. Blood glucose measurements were made at 15, 30, 60, and 120 min after carbohydrate loading. The area under the curve (AUC) for increase in glucose over baseline was calculated during OGT responses by incremental method. 

### Short-term studies

To assess the influence of OA and MA on the activity intestinal carbohydrate hydrolyzing enzymes and glucose transporters, groups of non-diabetic and STZ-induced diabetic male Sprague-Dawley rats were housed individually in Makrolon polycarbonate metabolic cages (Techniplats, Labotec, South Africa) for a 5-week period (n = 6 in each group). In those animals in which the effects of OA/MA were investigated, the rats were administered with OA/MA (80 mg/kg) twice daily at 09h00 and 15h00 by means of a bulbed steel tube. Rats similarly treated with DMSO/saline (3 mL/kg, p.o.) and standard anti-diabetic drugs (acarbose, 100 mg/ kg, p.o.) acted as untreated and treated positive controls, respectively. 

### Tissue sample harvesting

At the end of the 5 week experimental period, all animals were sacrificed by exposing to halothane via a gas anaesthetic chamber (100 mg/kg, for 3 min). The tail part of the pancreas was quickly removed from each rat through the abdominal incision. Thereafter, the whole of the small intestine was removed by cutting across the upper end of the duodenum and the lower end of the ileum from the pyloric sphincter to the ileocecal junction and rinsed with cold normal saline solution. Mid portions of the small intestine (10 ± 2 cm) were removed, snap frozen in liquid nitrogen and stored in a BioUltra freezer (Snijers Scientific, Tilburg, Netherlands) at -70 °C for Western blot analysis of glucose transporters, SGLT1 and GLUT 2 and carbohydrate hydrolyzing enzymes, α-amylase, α-glucosidase. 

### Western Blot Analysis

Small intestine tissues harvested from untreated OA and MA treated STZ-induced diabetic rats at the end of 5 week were analyzed for SGLT1, GLUT 2, α-amylase and α-glucosidase using Western blotting. Pancreatic tissues were only analyzed for α-amylase. Small intestine and pancreatic tissues (0.1 g) were homogenized on ice in isolation buffer (0.5 mM Na_2_EDTA, 0.1 M KH_2_PO_4_, 0.1 mM dithiothreitol, 0.25 M sucrose) and then centrifuged at 400 x g for 10 min (4 °C). The protein content was quantified using the Lowry method [17]. All the samples were standardized to one concentration (1 mg/mL). The proteins were then denatured by boiling in laemmli sample buffer (0.5 M Tris-HCl, glycerol, 10% sodium dodecyl sulphate (SDS), 2-mercaptoethanol, 1% bromophenol blue) for 5 min. The denatured proteins were loaded (25 µL) on prepared resolving (10%) and stacking (4%) polyacrylamide gels along with molecular weight marker (5 µL). The gel was electrophoresed for 1 h at 150 V in electrode (running) buffer (Trisbase, glycine, SDS), pH 8.3). Following electrophoresis, the resolved proteins were electro-transferred to an equilibrated polyvinylidene difluoride (PVDF)/ membrane for 1 h in transfer buffer (192 mM glycine, 25 mM Tris, 10% methanol). After transfer, the membrane was blocked with 5% non-fat dry milk in Tris-buffered saline with 0.1% Tween 20 (TTBS) (20 mM Tris, 150 mM NaCl, KCl, 0.05% Tween-20). 

The intestinal membranes were then immuno-probed with antibodies- SGLT1, GLUT 2, α-amylase and α-glucosidase (1:1000 in 1% BSA, Neogen, USA) while the pancreatic membranes were immune-probed with α-amylase (1:500 in 1% BSA, Neogen, USA) for 1 h at room temperature (RT). The PVDF membrane was then subjected to 5 washes (10 min each with gentle agitation) with TTBS. The membranes were then incubated in horse radish peroxidase (HRP)-conjugated secondary antibody (rabbit anti-mouse 1:10 000; Bio-Rad) for 1 h at RT. After further washing, antigen-antibody complexes were detected by chemiluminescence using the Immune-star™ HRP substrate kit (Bio-Rad, Johannesburg, South Africa). Chemiluminescent signals were detected with the Chemi-doc XRS gel documentation system and analysed using the quantity one software (Bio-Rad, Johannesburg, South Africa). Band intensity analysis was conducted on the resultant bands.

### In-vitro inhibitory enzyme assay studies

The inhibitory activities of OA and MA on α-amylase and *α*-glucosidase were studied using an α-amylase/*α*-glucosidase-starch model system while inhibitory effects on sucrose utilized the dextran sucrase- dextran sucrose reaction mixture.

### α-amylase

The assessment of the inhibitory effects of OA/MA against α-amylase activity *in vitro* was based on the modified method previously described Bhandari et al., 2008 and Gao et al., 2008 ([18,19]. Briefly, soluble maize starch (1 mg) was boiled for 5 min in 0.5 mL of 0.5M Tris-HCl buffer (pH 6.9) containing 0.01M CaCl_2_. After cooling, deionized water was added to a final volume of 100 mL. The solution was kept in the refrigerator and was used within 2-3 days. A reaction mixture, 500 μL containing 200 μL starch, 100 µL of OA at various concentrations (4.37-21.90 μmol/L) to which 200 μL of α-amylase (porcine pancreas, 2.60 mmol/L) was added to initiate the reaction and incubated at 37°C for 37 min. The reaction was terminated by addition of 100 μL of 50% acetic acid. 

### α-glucosidase

The assessment of the inhibitory effects of OA and MA against *α*-glucosidase *in vitro* utilized a similar method described above for α-amylase except that the 0.1M potassium phosphate buffer (pH 6.9) was used. The assay mixture (500 µL) comprising of 200 µL of α-glucosidase (Type 1, Bakers yeast, 1.30 mmol/L) was premixed with OA (100 µL) at various concentrations (4.37-21.90 μmol/L). The mixture was incubated at 37 °C for 30 min after adding starch in phosphate buffer and stopped by adding 1.5 mL of 2M Tris-HCl buffer (pH 6.9). 

### Sucrase

The *in vitro* sucrase inhibitory effects of OA/MA were performed as described above for α-amylase except for that the assay mixture (700 µL) comprised of 200 µL dextran sucrose (56 mmol/L) in the potassium phosphate buffer, 100 µl of OA/MA at various concentrations (4.37-21.90 μmol/L) and 400 μL of dextran sucrase (*Leuconostoc mesenteroides*, 2.60 mmol/L).

In all cases, the liberated glucose was measured by the glucose oxidase method and the absorbance was recorded at 595 nm using Varian Cary 1E UV-visible spectrophotometer (Varian Australia Pty Ltd, Mulgrave, Victoria, Australia). Results expressed as the percentage inhibition of the corresponding control were calculated using the formula:

$$\mathrm{\% Inhibition}=\frac{{\mathrm{Abs}}_{\mathrm{Control}}{\mathrm{-Abs}}_{\mathrm{Sample}}}{{\mathrm{Abs}}_{\mathrm{Control}}}\mathrm{X 100}$$

Where Abs_Control_ was the absorbance without sample,

Abs_samples_ was the absorbance of sample extract.

The IC_50_ values were calculated from plots of log concentration of inhibitor concentration versus percentage inhibition curves.

### Statistical analysis

Data are expressed as means ± SEM. Statistical significance was performed with GraphPad InStat Software (version 5.00, GraphPad Software, San Diego, California, USA), for analysis of one-way variance (ANOVA), followed by Tukey-Kramer multiple comparison test.

## Results

### Structure elucidation

The ^1^H- and ^13^C-NMR (1D and 2D) spectroscopic data for OA and MA obtained from *S. aromaticum* powder EAS white powder following recrystallization with chloroform-methanol were given as follows:

### OA

^1^HNMR CDCl_3_ δ, 0.73, 0.75, 0.88, 0.89, 0.90, 0.96, 1.11 (each 3H, s), 2.84 (1H, dd, *J* = 10.36), 3.22 (1H, dd, *J* = 4.56, 5.26 (1H, t, *J* = 3.76); ^13^C NMR (CDCl_3_) : δ 183.5, 143.8, 122.7, 79.2, 55.4, 47.8, 46.8, 46.1, 41.8, 41.2, 39.5, 38.9, 38.6, 38.3, 34.0, 33.3, 32.8, 32.7, 31.6, 28.4, 27.9, 27.4, 26.2, 23.8, 23.7, 23.1, 18.5, 17.4, 15.8, 15.5. 

**Figure 1:**
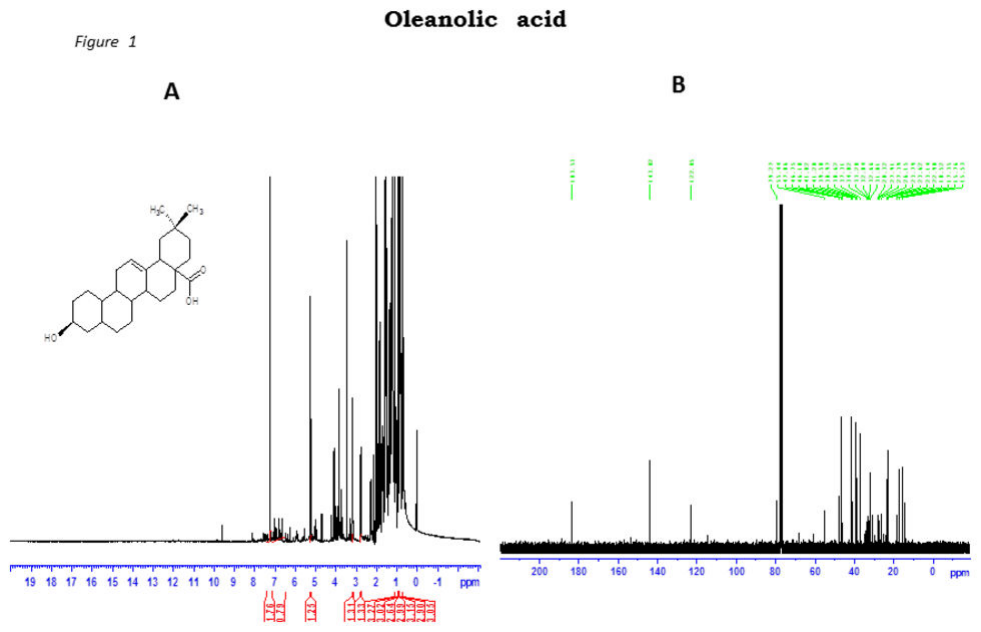
^1^H (A) and ^13^C (B) NMR of oleanolic acid in dissolved in chloroform.

These values compared with those reported in literature [20]. Spectra in Figure 1 are given in the supporting information. The purity of the plant-derived OA was approximately 98% and the percentage yield varied from 0.79% to 1.72%. 

### MA

^13^C NMR: δ_H_ (^13^C NMR (400MHz,CD3OD): 46.2 (C-1), 68.3 (C-2), 83.3 (C-3), 39.1 (C-4), 55.0 (C-5), 18.1 (C-6), 32.7 (C-7), 39.0 (C-8), C-9 (47.4), C-10 (38.0), C-11 (23.2), C-12 (121.9), C-13 (143.7), C-14 (41.6), C-15 (27.4), C-16 (23.0), C-17 (46.2), C-18 (41.0), C-19 (45.7), C-20 (30.4), C-21 (33.6), C-22 (32.3), C-23 (28.3), C-24 (16.6), C-25 (16.5), C-26 (16.4), C-27 (23.2), C-28 (178.5), (C-29) 32.2, C-30 (23.2). 

**Figure 2:**
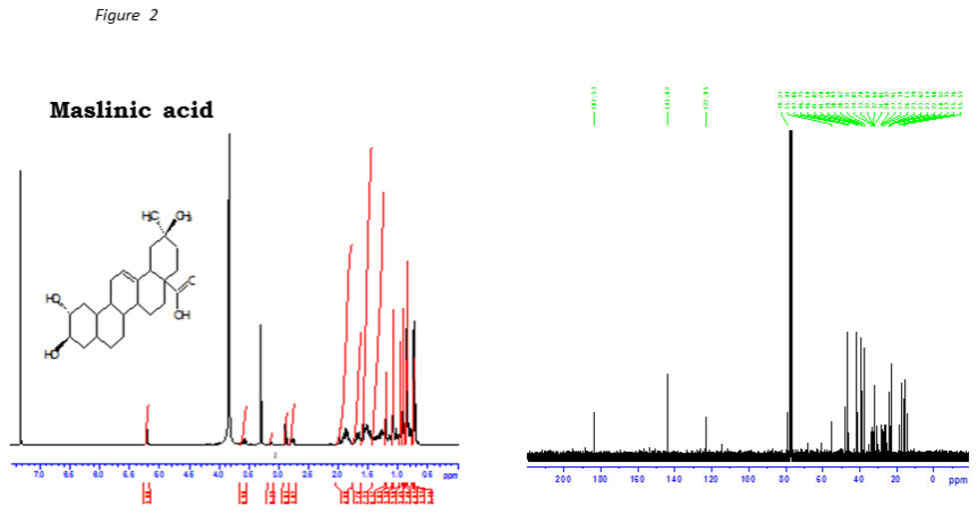
^1^H (A) and ^13^C (B) NMR spectroscopic analysis of MA dissolved in deuterated methanol.

These data compared with those reported in literature [3]. Spectra in Figure 2 are given in the supporting information. The purity of the plant-derived MA was approximately 98% and the percentage yield varied from 0.02% to 0.03%. 

### Postprandial glucose concentrations

**Figure 3:**
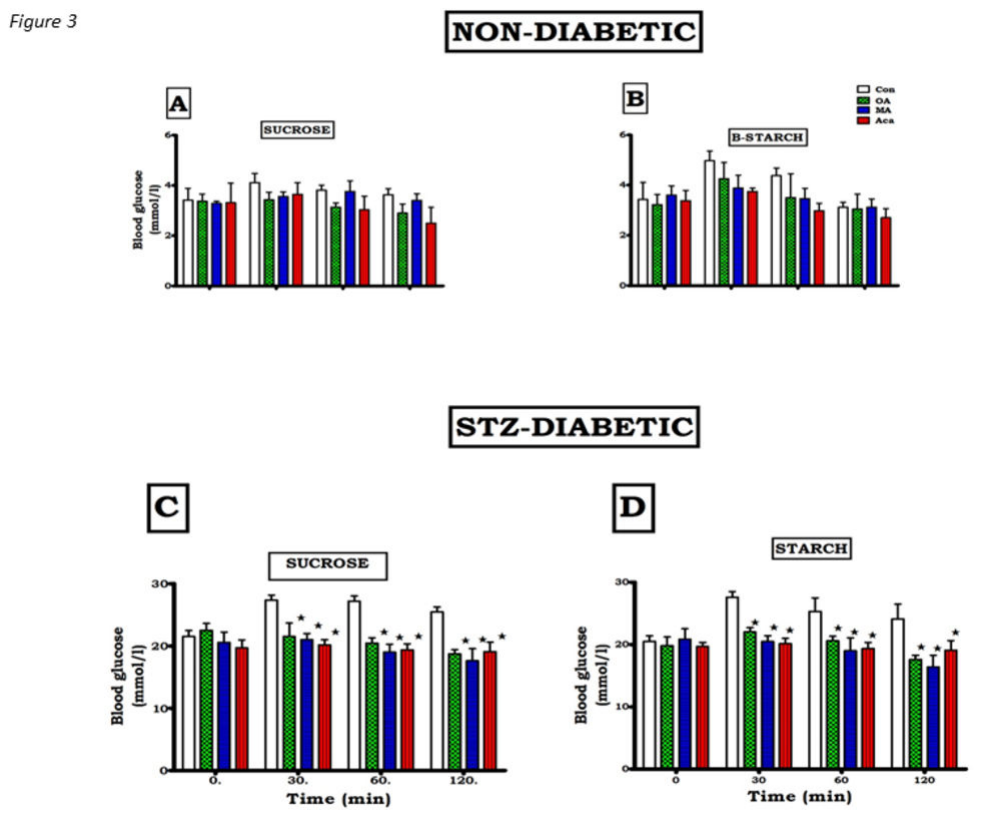
Comparison of OGT responses to OA and MA in non-diabetic (A and B) and STZ- induced diabetic (C and D) rats after sucrose and starch loading. Values are presented as means, and vertical bars indicate SEM (n=6 rats in each group). ★ p<0.05 by comparison with control animals.

**Figure 4:**
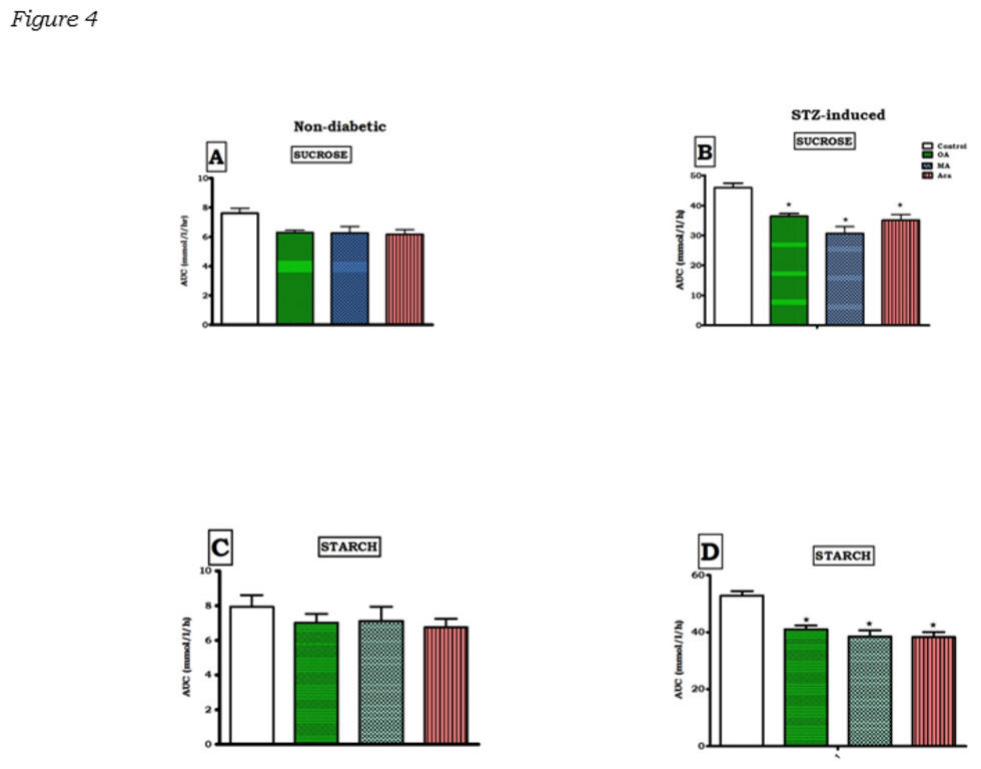
Inhibitory effects of OD and MA on blood glucose increases in non-diabetic (A and B) and STZ-induced diabetic (C and D) rats after sucrose and starch loading for 2h. The area under the curve for glucose (AUC_glucose_) was calculated by incremental method. Values are presented as means, and vertical bars indicate SEM (n = 6 rats in each group). ★p < 0.05 by comparison with control animals.

Blood glucose concentrations were significantly higher in the STZ-induced diabetic rats groups loaded with sucrose and at all time-points during the OGT response tests compared to non-diabetic rats (Figure 3). The glucose area under the curve (AUC) also increased significantly compared to non-diabetic rats (Figure 4). Treating STZ-induced diabetic rats with OA significantly reduced blood glucose levels during the OGT protocol. In addition, the AUC_glucose_ was smaller in treated diabetic animals compared with that in untreated diabetic rats. In the MA-treated diabetic rats, the OGT responses and AUC_glucose_ were not significantly different from those observed with OA. Similar kind of suppression effect was observed in the diabetic groups that received acarbose as the positive control along with the carbohydrates. Considering the whole 120 min experiment, the glycaemic responses of STZ-induced diabetic rats to sucrose or starch co-administration with OA/MA were not significantly different. 

The blood glucose concentrations of control non-diabetic rats increased slightly after carbohydrate load, but co-administration with OA and MA decreased the blood glucose levels to values that did not achieve statistical significance (Figure 3). This was reflected the AUC_glucose_ of groups of animals administered with the triterpenes that did not achieve statistical difference when compared to control non diabetic rats (Figure 3). 

### Western blots

**Figure 5:**
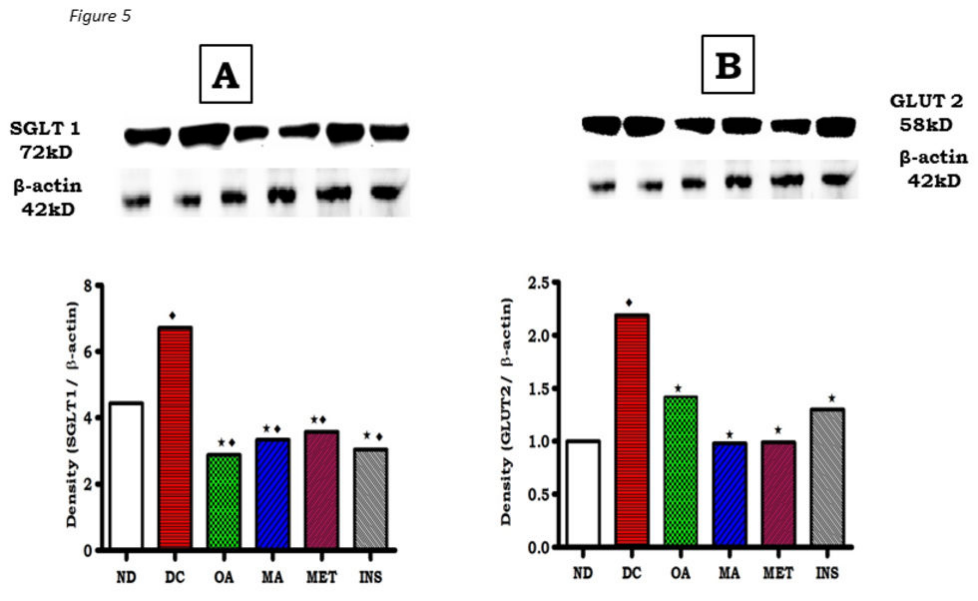
Effects of OA and MA on expression of SGLT1 (A) and GLUT2 (B) as determined by Western blotting of control, OA, MA, metformin and insulin -treated small intestine tissues of non-diabetic and STZ-induced diabetic rats. Values are expressed as mean ± S.E.M. Values were obtained from Western blots for six preparations. ★ p <0.05 by comparison with respective control animals. ◆ p <0.05 by comparison with respective non-diabetic animals.

Western blot analysis of glucose transporters SGLT1 and GLUT2 and carbohydrate hydrolyzing enzymes, α-amylase, α-glucosidase proteins in small intestines isolated from control and treated non-diabetic and STZ-induced diabetic rats were assessed to explore the inhibitory mechanism of OA and MA on glucose absorption. The small intestines revealed that STZ-induced diabetes increased the expression of SGLT1 and GLUT2 proteins in the intestines of diabetic rats by 43% and 23%, respectively compared to non-diabetic control rats (Figure 5). The increases of SGLT1 and GLUT2 induced in diabetes were significantly abrogated by OA and MA indicating that the triterpenes reduced small intestine glucose absorption in part via inhibition of glucose transporters. Interestingly, treatment with OA and MA reduced the SGLT1 expression in small intestines isolated from diabetic rats to values significantly lower than those of untreated diabetic and non-diabetic control rats, but comparable to the standard hypoglycaemic drugs (insulin and metformin). 

**Figure 6:**
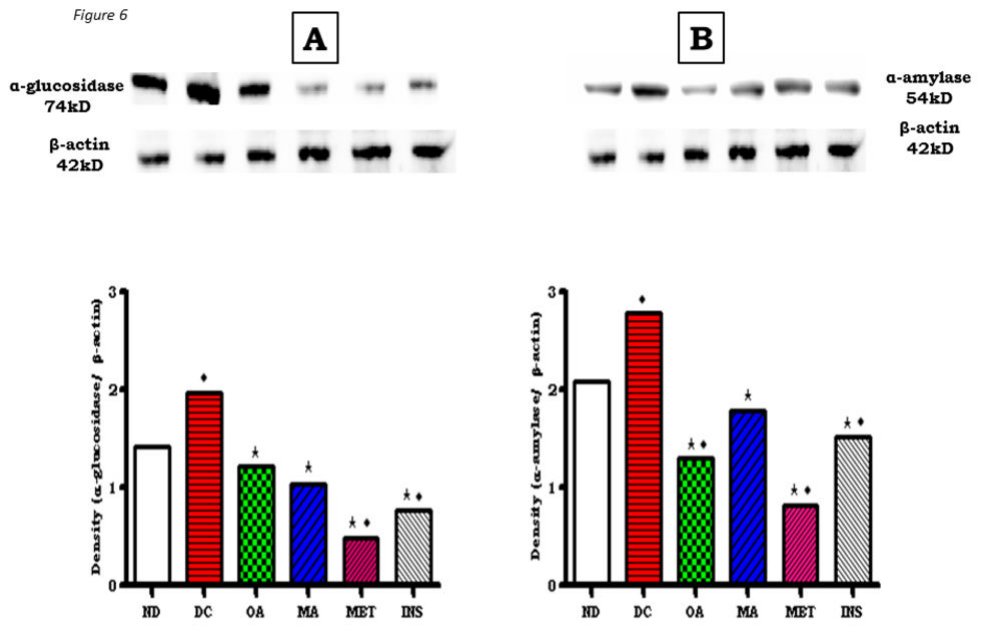
Effects of OA and MA on expression of α-glucosidase (A) and α-amylase (B) as determined by Western blotting of control, OA, MA, metformin and insulin-treated small intestine tissues of non-diabetic and STZ-induced diabetic rats. Values are expressed as mean ± S.E.M. Values were obtained from Western blots for six preparations. ★ p <0.05 by comparison with respective control animals. ◆ p <0.05 by comparison with respective non-diabetic animals.

To determine whether these effects also involved intestinal carbohydrate hydrolyzing enzymes, the levels of α-amylase and α-glucosidase were determined using Western blot analysis in small intestines isolated from control and treated non-diabetic and STZ-induced diabetic rats. As assessed by densitometric analyses, α-amylase and α-glucosidase levels in untreated diabetic rats were elevated in comparison with non-diabetic control rats. OA significantly reduced the expression of both α-amylase and α-glucosidase of STZ-induced rats, but effects were more potent on the former enzyme (Figure 6). MA like OA inhibited the expression of both enzymes with no significant difference in potency.

**Figure 7:**
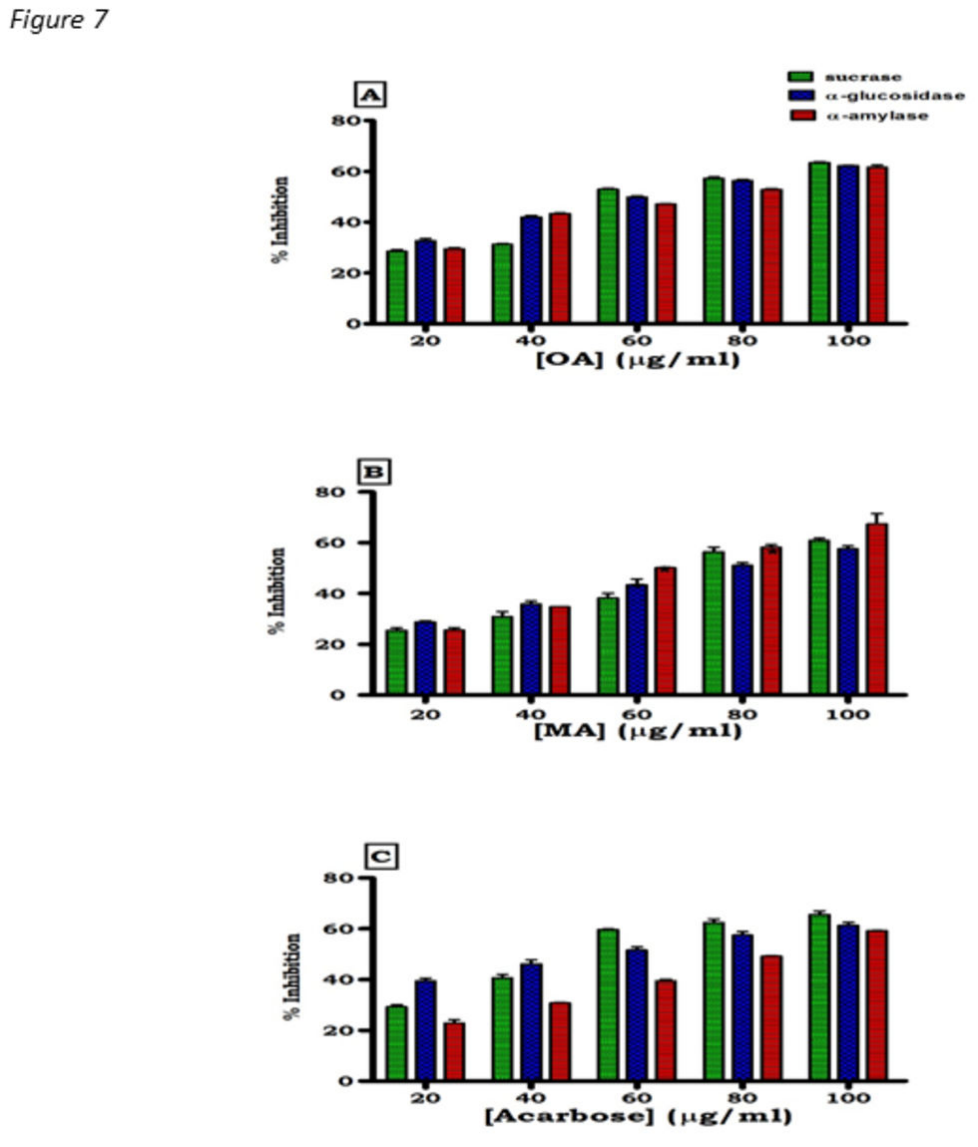
Percent inhibition by OA (A), MA (B) and acarbose (C) sucrase on dextran sucrose and of α-glucosidase and α-amylase on starch. Values presented are means ± SEM (*n* = 8 for each concentration).

**Figure 8:**
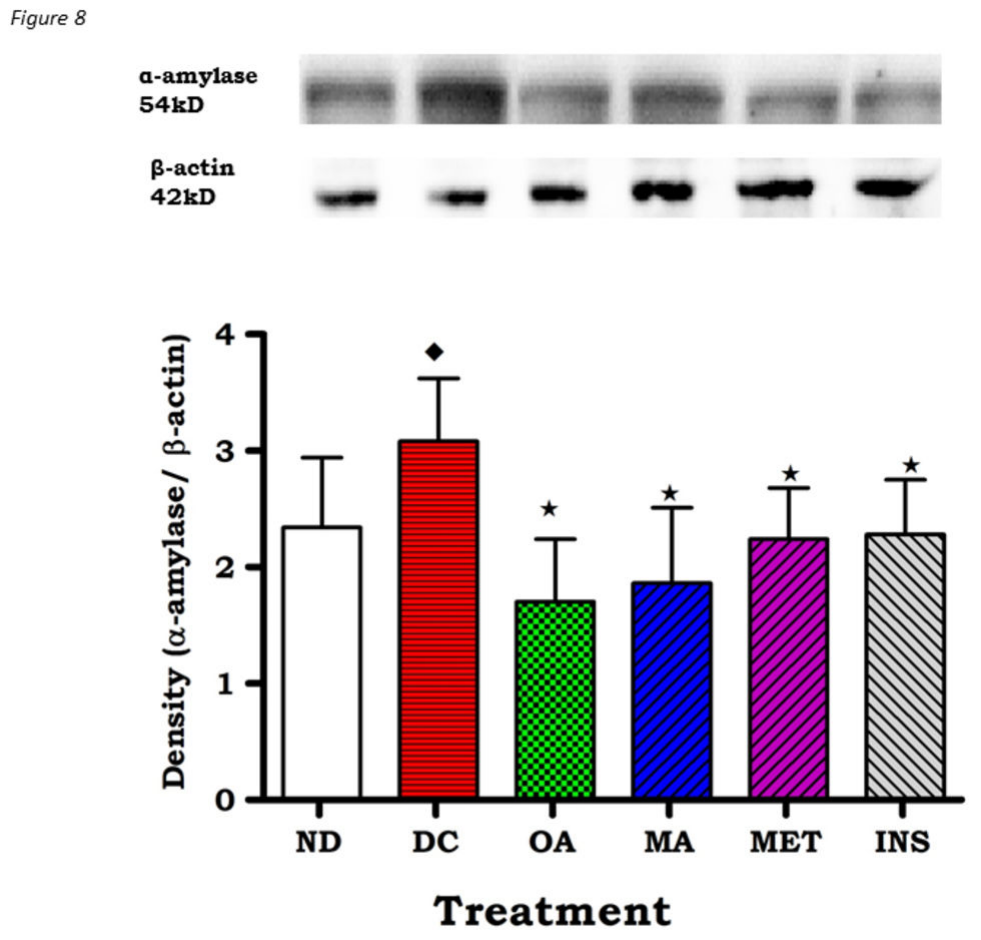
Effects of OA and MA on expression of α-amylase as determined by Western blotting of control, OA, MA, metformin and insulin -treated pancreatic tissues of non-diabetic and STZ-induced diabetic rats. Values are expressed as mean ± S.E.M. Values were obtained from Western blots for six preparations. ★ p <0.05 by comparison with respective control animals. ◆ p <0.05 by comparison with respective non-diabetic animals.

To further explore these findings, we used *in vitro* assays to quantify the inhibitory activities of OA and MA against α-amylase, α-glucosidase and sucrase. Both OA and MA dose dependently inhibited α-amylase, α-glucosidase and sucrase activities (Figure 7). By comparison with the standard anti-amylase drug acarbose, IC_50_ values of OA against α-amylase, α-glucosidase and sucrase of 13.60 ± 0.18 vs 17.50± 0.17, 10.40 ± 0.11vs 15.50 ± 0.14 and 10.50 ± 0.13 vs 13.10 ± 0.17 mmol/L, respectively, were significantly low. The MA IC_50_ values against these key carbohydrate hydrolyzing enzymes did not significantly differ from those of OA. The effects of diabetes and subsequent OA and MA treatments on α-amylase expression in the pancreas were also evaluated in STZ-induced diabetic rats. As evaluated by Western blot analysis, pancreatic tissues from untreated STZ-induced diabetic rats exhibited significant increases (p<0.05) in pancreatic amylase expression compared to non-diabetic control animals (Figure 8) which was significantly (p<0.05) towards control values in non-diabetic rats after OA and MA treatment. The reversal of these changes by the triterpenes was similar to those changes that occurred following standard drug treatments.

## Discussion

The present study was designed to evaluate whether *Syzigium aromaticum*-derived triterpenes, OA and MA reduce postprandial glucose in STZ-induced diabetic rats loaded with carbohydrates via inhibition of carbohydrate hydrolysis and reduction of glucose transporters in the gastrointestinal tract. The structures of OA and MA were elucidated by ^1^H and ^13^C NMR spectral data compared with previously reported values [3,20]. We have previously reported that the hypoglycaemic properties may arise, in part, from the inhibition of glucose transport across the small intestine [12]. The results suggest that OA and MA inhibit the activity of key carbohydrate hydrolyzing enzymes and down regulate the activity of glucose transporters in the small intestines to decrease postprandial hyperglycaemia. 

OA and MA improved glycaemic control of STZ-induced diabetic rats as evidenced by the suppression of postprandial glucose concentration and reduction in the AUC_glucose_. The inhibition of the α-amylase and α-glucosidase as well as down regulating of glucose transporters in the small intestines by the triterpenes possibly contributed to the decrease in postprandial glucose [21,22]. Interestingly, intestinal α-glucosidase enzyme inhibitors also improve postprandial hyperglycaemia by delaying digestion of polysaccharides, disaccharides and monosaccharaides [21,23,24]. Pancreatic α-amylase and intestinal α-glucosidase hydrolyze the α-1, 4-glucoside linkages carbohydrates to glucose which is then transported through the mucosa of the bowel [25]. On the other hand, SGLT1 in the apical membranes of the intestinal mucosa reabsorbs glucose from the lumen across the brush border membrane which is released into the bloodstream via basolaterally expressed GLUT2 [26,27]. Therefore, the extend our previous observations by showing that the hypoglycaemic effects of triterpenes are in part mediated via inhibition of carbohydrate hydrolyzing enzymes as in the small intestines of diabetic rats. We have previously shown that triterpenes reduce blood glucose concentrations of STZ-induced diabetic rats via a variety of mechanisms [12-14].

Western blot analysis not only confirmed the inhibitory effects of OA and MA against α-glucosidase and pancreatic α-amylase, but additionally showed that the triterpenes may potentially reduce glucose absorption by decreasing the levels of SGLT and GLUT2 in the small intestines of STZ-induced diabetic rats. It has been shown in rats with experimentally induced diabetes that the capacity of the small intestine to absorb glucose increases at least in part, due to enhanced activity and abundance of brush border SGLT1 and basolateral GLUT2 [5]. Therefore, it is possible that down regulation of these transporters by OA and MA reduces the total glucose absorption capacity in the small intestine in diabetes. Indeed, untreated STZ-induced diabetic rats exhibited increased SGLT1 and GLUT2 expression in the small intestine in comparison to non-diabetic control animals.

Interestingly, *in vitro* studies indicated that the IC_50_ values of OA and MA against sucrase, α-amylase and α-glucosidase at various concentrations were less than those shown of acarbose suggesting that the triterpenes have fewer side effects. Acarbose is associated with gastrointestinal side effects due to excessive inhibition of pancreatic α-amylase, resulting in the abnormal bacterial fermentation of undigested carbohydrates in the large intestine [28,29]. Additionally, acarbose has been reported to increase the incidence of renal tumors, serious hepatic injury and acute hepatitis [30,31]. Against this background are reports that several *α*-glucosidase inhibitors isolated from medicinal plants possess lesser adverse effects than the existing drugs [11,32-34]. 

Altogether, it can be suggested that the OA and MA modulates the activity of intestinal glucose transporters and carbohydrate hydrolyzing enzymes to reduce postprandial hyperglycaemia in diabetes. The reduction by OA and MA of postprandial hyperglycaemia and the AUC_glucose_ are characteristics of effective compounds which control diabetes [35,36]. The data suggest that OA and MA could be used as a potential supplements for treating postprandial hyperglycemia.
